# Novel Ligands for a Purine Riboswitch Discovered by RNA-Ligand Docking

**DOI:** 10.1016/j.chembiol.2010.12.020

**Published:** 2011-03-25

**Authors:** Peter Daldrop, Francis E. Reyes, David A. Robinson, Colin M. Hammond, David M. Lilley, Robert T. Batey, Ruth Brenk

**Affiliations:** 1 Division of Biological Chemistry and Drug Discovery (BCDD), College of Life Sciences, University of Dundee, Dow Street, Dundee DD1 5EH, UK; 2 Department of Chemistry and Biochemistry, University of Colorado, Boulder, CO 80309-0215, USA; 3 CR-UK Nucleic Acid Structure Research Group, MSI/WTB complex, University of Dundee, Dow Street, Dundee DD1 5EH, UK

## Abstract

The increasing number of RNA crystal structures enables a structure-based approach to the discovery of new RNA-binding ligands. To develop the poorly explored area of RNA-ligand docking, we have conducted a virtual screening exercise for a purine riboswitch to probe the strengths and weaknesses of RNA-ligand docking. Using a standard protein-ligand docking program with only minor modifications, four new ligands with binding affinities in the micromolar range were identified, including two compounds based on molecular scaffolds not resembling known ligands. RNA-ligand docking performed comparably to protein-ligand docking indicating that this approach is a promising option to explore the wealth of RNA structures for structure-based ligand design.

## Graphical abstract

**Graphical Abstract:**

**Figure:**
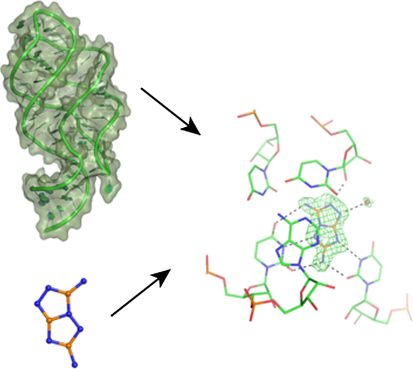

**Highlights:**

► Using RNA-ligand docking, four new ligands were discovered for a purine riboswitch ► Two of the ligands were based on scaffolds not known to bind to this riboswitch ► Crystal structures were determined that confirm the binding modes of new ligands ► Molecular docking is a promising method for RNA-structure-based ligand design

## Introduction

Target-driven drug discovery efforts have focused traditionally on modifying protein functions. By contrast, RNA has remained largely unexplored as a drug target. Despite its polyelectrolyte character, RNA can adopt intricate three-dimensional structures that are essential for function. The folded structure enables RNA to bind small molecules with high affinity and selectivity and even to catalyze chemical reactions (Holbrook, 2005). RNA plays a central role in almost every genetic process of the cell, and is therefore a potential drug target (Hermann, 2000; Blount and Breaker, 2006; Thomas and Hergenrother, 2008). Indeed, many long-known antibiotics bind to the 16S ribosomal RNA component of the bacterial ribosome (Moazed and Noller, 1987). More recently, it was discovered that also sections of some mRNAs (termed riboswitches) are targets for antibiotics (Blount and Breaker, 2006; Kim et al., 2009; Lee et al., 2009; Ott et al., 2009; Mulhbacher et al., 2010). The increasing number of RNA crystal structures enables a structure-based approach to the discovery of novel RNA-binding ligands (Franceschi and Duffy, 2006; Schwalbe et al., 2007; Serganov, 2010). However, whereas structure-based techniques are routinely used in the protein field, their application in the RNA field is still in its infancy (Fulle and Gohlke, 2010).

One way to exploit the target structure for ligand binding is fragment screening, which has recently been applied to RNA targets (Bodoor et al., 2009; Chen et al., 2010). Molecular docking is another key method in the area of structure-based design (Klebe, 2006). Docking predicts the preferred orientation of a small molecule in binding to a receptor to form a stable complex. Docking can also be used for virtual screening of databases containing millions of molecules for potential ligands. In that case, each database entry is sequentially docked into the binding site and scored for its fit, resulting in a score-ranked list.

There are two strategies for RNA-ligand docking: (1) to adopt methods and scoring functions originally developed for protein-ligand docking (Lind et al., 2002; Detering and Varani, 2004; Kang et al., 2004; Moitessier et al., 2006; Park et al., 2008; Lang et al., 2009; Li et al., 2010b) and (2) to develop entirely new scoring functions or docking algorithms (Morley and Afshar, 2004; Barbault et al., 2006; Pfeffer and Gohlke, 2007; Guilbert and James, 2008). What all of these RNA docking studies have in common is that the vast majority of the investigated targets are rather complex including large and flexible ligands, water-mediated interactions, and flexible receptors. All these approaches are still challenging for the more-explored protein-ligand docking (Klebe, 2006) and make it difficult to disentangle these effects from issues specific to RNA-ligand docking. In order to develop this field, we therefore sought a model system that would be both experimentally and computationally tractable, and that would facilitate the dissection of various contributions to ligand binding energies to guide improvement of computational methods. In this study we have selected the *Bacillus subtilis xpt-pbuX* guanine riboswitch carrying a C74U mutation (called GRA) (Gilbert et al., 2006b) as such a model system, and we demonstrate its potential for probing RNA-ligand docking.

Riboswitches are *cis*-acting gene regulatory elements that are mostly found in bacteria (Lee et al., 2009). They are located in the 5′ untranslated region (UTR) of mRNAs and consist of an aptamer domain that binds the ligand, and an expression platform that controls the expression of the downstream gene. The RNA can adopt one of several alternative conformations, the relative stability of which is determined by the binding of the ligand to the aptamer domain. Binding of the ligand directs folding of downstream elements in the expression platform that influence expression. Thus, regulation of gene expression is controlled by the concentration of the small molecule ligand via the structure of the RNA. Crystal structures of over ten riboswitches have been determined, making this class of RNA amenable for structure-based drug design (Schwalbe et al., 2007; Serganov, 2010).

**Figure 1:**
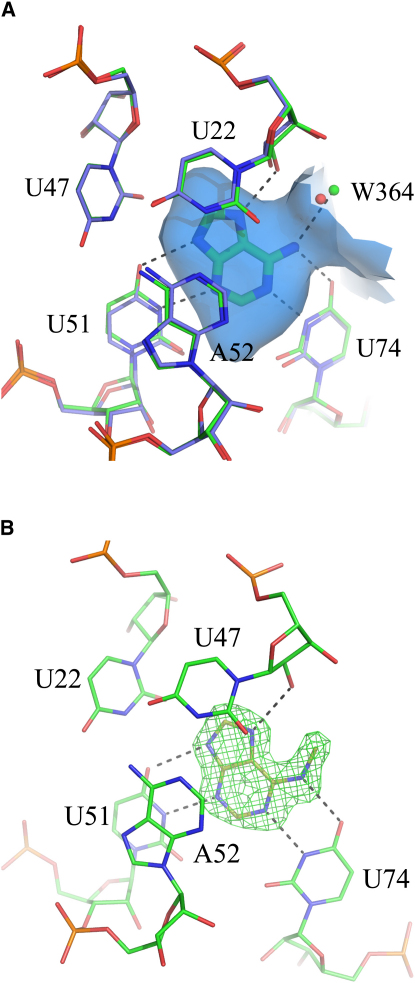
Binding Sites of the Adenine and the *B. subtilis* xpt-pbuX Guanine Riboswitch C74U Mutant (A) Binding pocket of the *V. vulnificus* AR bound to adenine (green carbon atoms; water molecule close to ligand as green sphere) superimposed with GRA (blue carbon atoms, water molecule close to bound ligand drawn as red sphere, ligand removed for clarity). The solvent accessible surface is shown in blue. Hydrogen bonds are marked as dashed lines. The nucleotides forming the binding pockets are fully conserved between AR and GRA and adopt the same conformation in the ligand bound structure. (B) Crystallographically determined binding mode of **15** together with *Fo-Fc* map (contoured on 3.0 σ) which was calculated by omitting the ligand from the final model. Putative hydrogen bonds are marked as dashed lines. The water molecule W364 from 2G9C is displaced by the N6-methyl group of the ligand.

The adenine-binding riboswitch (AR) and the related GRA are among the best characterized riboswitches. The GRA was derived by a point mutation from the guanine riboswitch (C74U) (Gilbert et al., 2006b). As a result, the specificity of the guanine riboswitch was altered to be adenine responsive, thus generating a new adenine riboswitch (GRA). Crystal structures have been determined for the aptamer domains of *Vibrio vulnificus* AR and *B. subtilis* GRA (Serganov et al., 2004; Gilbert et al., 2006a). The bases of both aptamer domains that form the binding site are fully conserved and adopt the same conformation in the ligand- bound structure (Figure 1A) (Serganov et al., 2004). The adenine binding site is rather small (108 Å^3^) and 98% shielded from bulk solvent. In the available crystal structures, all ligands are bound in a similar orientation, forming multiple hydrogen bonds with the nucleotides U22, U51, as well as U74, and pi-stacking interactions with U47, A52, A21, and U75. A conserved water molecule located toward the opening of the pocket forms an additional hydrogen bond to the ligands. Binding studies with both AR and GRA have been carried out and a range of ligands as well as compounds not binding to these riboswitches (here referred to as decoys) have been identified (Mandal and Breaker, 2004; Gilbert et al., 2006a, 2009).

The program DOCK 3.5.54 was applied to dock ligands into the riboswitch structure (Lorber and Shoichet, 1998; Wei et al., 2002). DOCK 3.5.54 uses a force-field-based scoring function to estimate the binding energy (E) that accounts for van der Waals (E_vdw_) and electrostatic energy (E_elec_) and corrects for the desolvation energy of the ligands (ΔG_solv_) when transferred from aqueous solution into the binding site (Equation 1):

(1) $$\text{E}={\text{E}}_{\text{elec}}+{\text{E}}_{\text{vd}\text{w}}+\Delta {\text{G}}_{\text{solv}}.$$

Unlike regression- or knowledge-based scoring functions, force-field-based scoring function are derived from physicochemical theory, and therefore do not require a training set of protein- or RNA-ligand complexes together with affinity data for parameterization (Gohlke and Klebe, 2001). Thus, the only modification required to adapt the DOCK 3.5.54 scoring function for RNA-ligand docking was to use RNA-specific parameters to calculate E_vdw_ and E_elec_. Docking was evaluated retrospectively using previously published data in terms of binding mode prediction, separation of known ligands and decoys, and enrichment of ligands among a database of similar compounds. For prospective predictions, high scoring compounds from a database screen were purchased and tested for ligand binding. Crystal structure complexes with selected ligands were determined to test the predicted binding modes. The implications of the results in respect to advancing RNA-ligand docking, molecular docking in general, and molecular recognition properties of riboswitches which bind adenine with high affinity are discussed.

## Results

### Retrospective Docking Results

#### Binding Mode Prediction

At the outset of the study, three crystal structures of riboswitches capable of binding adenine with high affinity were known: *V. vulnificus* AR in complex with adenine (**4** in Table 1) and *B. subtilis* GRA in complex with 2,6-diaminopurine (**1**) and 2,4,6-triamino-pyrimidine (**5**) (Serganov et al., 2004; Gilbert et al., 2006a, 2006b). The binding sites of AR and GRA are fully conserved and the conformation of the binding site residues in all crystal structures were the same within experimental error (rmsd <0.3 Å, Figure 1A). All structures included a water molecule hydrogen bonded to the ligand. This water molecule was therefore retained for all docking calculations. The GRA structure in complex with pyrimidine-2,4,6-triamine, which has the highest resolution among the three riboswitch structures (1.7 Å), was chosen as receptor for docking. Hydrogen atoms were added and their geometry optimized. Partial charges for RNA atoms were obtained from the AMBER99 force field (Wang et al., 2000) except that the charge of the phosphate oxygen atoms was increased to yield a net charge of zero per nucleotide as done previously (Detering and Varani, 2004; Moitessier et al., 2006). Subsequently, all three ligands for which binding modes were known were docked into the receptor using DOCK 3.5.54. Comparison of the predicted binding modes with those determined crystallographically revealed that each was close to native (rmsd <0.34 Å, Table 1).

### Separation of Known Ligands and Decoys

Next, we tested whether a separation of decoys and ligands could be achieved by docking. A test set of compounds with known binding properties for AR and GRA was compiled (Mandal and Breaker, 2004; Gilbert et al., 2006a). For three ligands it was shown that they bind with comparable binding affinities to both riboswitches (Gilbert et al., 2006a). We therefore assumed that binding data for these riboswitches was transferable and included ligands and decoys determined with either riboswitch variant in the test. Conflicting results were reported for guanine. Whereas for AR a binding affinity of >10 μM was determined, no binding to GRA could be observed for this compound (Mandal and Breaker, 2004; Gilbert et al., 2009). The last result is in agreement with the established pharmacophore for AR ligands (Mandal and Breaker, 2004). Therefore, guanine was classified as decoy for this study. In total, the test set contained 23 compounds, 8 ligands, and 15 decoys (Table 1). The binding affinities of the ligands ranged from 0.01 to 100 μM. For the decoys, no binding was detected up to 300 μM except for guanine which was tested up to its solubility limit.

All compounds in the test set were docked into the GRA binding site and sorted by their score (Table 1). A clear separation of ligands and decoys was obtained. Seven out of the eight top-scoring compounds were true ligands. The probability to achieve such a distribution by chance is only 0.02%. All eight compounds with the lowest score were decoys.

The highest scoring decoy, 8-azaadenine (**6**), ranked sixth. In the docking database, this compound was stored as a neutral molecule. However, the pK_a_ of the hydrogen atom bearing N1 atom in this compound was calculated to be 7.6. Accordingly the compound is predominantly deprotonated under assay conditions (pH 8.3). When the negatively charged species was docked into the receptor, this compound received a score of only +15.52 kJ/mol, ranking at 22.

N6-methyladenine (**15**) was the weakest ligand in the test set. It obtained the least favorable score of all ligands, even lower than many of the decoys (Table 1). However, **15** is also the largest molecule of all binders and thus requires the largest amount of space in the binding pocket. In the predicted binding mode, the ligand adopts a conformation distinct from adenine with the N6-methylamino group pointing into the cleft between U51 and U74 resulting in unfavorable interactions (not shown). In order to adopt a similar binding mode as adenine the structural water molecule W364 would have to be expelled from the receptor (Figure 1A). This prompted us to determine the crystal structure of the N6-methyladenine-riboswitch complex (Table 2). The crystallographic analysis revealed that **15** indeed expels W364 from the binding site and adopts a similar binding mode as adenine (Figure 1B). When docked into the receptor without the water molecule present the correct binding mode was obtained (rmsd = 0.5Å). With a score of −32.83 kJ/mol the ligand would rank third in the database screen.

The three compounds in the test set with submicromolar binding constants ranked among the top five highest scoring compounds (Table 1). Yet despite this, no strong correlation between binding affinities and scores was found (Pearson correlation coefficient r = 0.64). This did also not improve when the corrected scores for **6** and **15** were taken into consideration (r = 0.68).

### Enrichment of Ligands from a Large Database of Similar Compounds

In the final retrospective experiment, we tested how well RNA-ligand docking can enrich the known ligands among the top scoring compounds when a large database was docked into the GRA binding site. For that purpose, we assembled a database with commercially available compounds. To avoid artificial enrichment (Verdonk et al., 2004) only compounds resembling the ligands and decoys in the test set were selected for this database. Our in-house database containing commercially available compounds (Brenk et al., 2008) was filtered for compounds containing up to 18 nonhydrogen atoms, one or two ring systems, at least one hydrogen bond donor and acceptor and a net charge between −1 and +2. In addition, the compounds had to be small enough to fit into the GRA binding site without causing a steric clash. The final database contained 2592 unique compounds.

**Figure 2:**
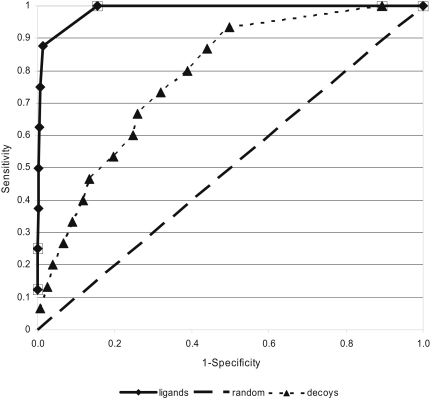
Receiver Operation Characteristic Plot for Ligands and Decoys in Database Screen The sensitivity (fraction of known compounds, ligands or decoys) was plotted against 1- specificity (fraction of unassigned database compounds). An AUC of 0.98 for ligands (solid lines) and 0.75 for decoys (dotted line) was obtained. A random prediction would result in an AUC of 0.5 (dashed line).

The compounds in the database together with those of the test set were docked into the GRA binding site and ordered by score. Based on this list the true positive rate (fraction of known compounds, ligands or decoys) was plotted against the false positive rate (fraction of unassigned database compounds) to obtain a receiver operation characteristic (ROC) curve (Figure 2) (Jain, 2004). The area under the curve (AUC) of a ROC curve is a measure of the test accuracy. A perfect prediction would be indicated by an AUC of 1.0 while random prediction would result in an AUC of 0.5. For the ligands an AUC value of 0.98 was obtained and for the decoys a value of 0.75. Thus, close to perfect enrichment of the ligands was achieved. The decoys were also enriched compared to random. The reason for this is that the decoys were selected on a rational basis to resemble known ligands and such have at least some of the properties required to bind into the GRA pocket (Mandal and Breaker, 2004).

### Prospective Results

Encouraged by the retrospective docking results we then performed more rigorous prospective tests. We selected a number of compounds for experimental characterization from among the top scoring hits obtained by docking the database of commercially available compounds. The selected compounds were either analogs of known ligands (**25**–**27**, Table 3) or compounds with novel scaffolds (**24** and **28**). The compounds were characterized both in terms of binding affinities and binding modes.

### Binding Affinities

**Figure 3:**
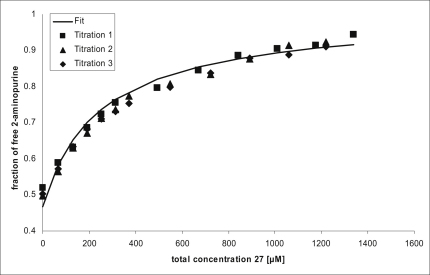
Plot of Fraction of Free 2-Aminopurine against Varying Concentrations of Compound **27** The data were fitted to a one binding site model with two equilibria. Three titrations are shown together with the resulting fit.

Two different methods were used to determine binding to the purine riboswitch in the past: in-line probing (Mandal and Breaker, 2004) and isothermal titration calorimetry (ITC) (Gilbert et al., 2006b). The in-line probing assay suffers from being an indirect method whereas for the ITC assay large quantities of RNA are required. Therefore, an alternative fluorescence assay was developed to determine the binding affinities of the putative ligands. Upon binding to GRA the fluorescence of 2-aminopurine (**3**, Table 1) decreases (Gilbert et al., 2006a; Lemay et al., 2006). The increase in fluorescence on displacement of 2-aminopurine was then studied as a function of the concentration of the putative ligand, using a constant concentration of 2-aminopurine and the riboswitch (Figure 3). The ITC assay and the fluorescence assay deliver comparable results. For **5** a binding constant of 0.020 mM was reported using the ITC assay (Gilbert et al., 2006b) while using the fluorescence assay a binding constant of 0.014 ± 0.001 mM was obtained. The amount of RNA used for a fluorescence competition experiment is less than 0.5% of that used for a typical ITC experiment.

Four out of the five chosen compounds bound to GRA with affinities in the micromolar range (Table 3). The most potent compound was **27** with a binding affinity of 80 μM. Addition of a methyl group (**26**) did not alter the binding affinity significantly (*K_D_* = 110 μM). When the methyl group was replaced by chlorine (**25**) no binding could be detected at concentrations up to 120 μM. Both compounds containing new scaffolds (**24** and **28**) were weak ligands with binding affinities of 370 and 670 μM, respectively.

### Binding Modes

**Figure 4:**
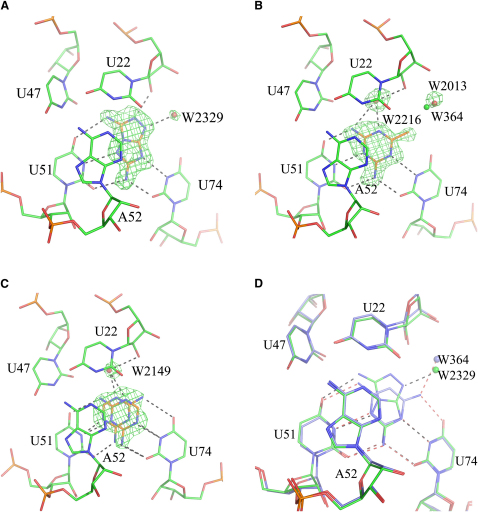
Crystallographically Determined Binding Modes for Ligands Identified by Molecular Docking Crystallographically determined binding modes of **24** (A), **26** (B), and **27** (C) together with *Fo-Fc* map (contoured on 2.5 σ for A and C and 3.0σ for B) which was calculated by omitting the ligands and water molecules from the final model. Putative hydrogen bonds are marked as dashed lines. In (B), the position of W364 from 2G9C is marked as green sphere. (D) Superposition of crystallographically determined binding modes of **2** (blue carbon atoms, water molecule W364 indicated as blue sphere) and **24** (green carbon atoms, water molecule W2329 indicated as green sphere). Hydrogen bonds are marked as dashed lines in red for **2** and black for **24**.

To determine the binding modes of the new ligands, we attempted cocrystallization with GRA. Crystallization trials with **28** led only to microcrystals of insufficient quality for data collection. However, high-resolution crystal structures were obtained with compounds **24**, **26**, and **27** (Table 2). The crystals diffracted up to 1.5 Å which is comparable to or better than previous crystal structures (Gilbert et al., 2006a, 2006b, 2009). The overall structure of the riboswitch was unchanged in all three crystal structures (rmsd values <0.4 Å compared to 2G9C). The ligands were clearly defined in the *Fo-Fc* electron density maps (Figure 4).

Compound **24** hydrogen bonds with the bases of U51 and U74, the ribose of U22 and water molecule W2329 which is located in an equivalent position to W364 in the docking calculations (Figure 4A). The observed and the predicted binding mode agreed within experimentally error (rmsd = 0.21 Å).

The crystal structure of GRA in complex with **26** showed clearly defined electron density for a ligand in the *Fo-Fc* map (Figure 4B). However, due to ligand symmetry the orientation cannot be inferred unambiguously from the map. Therefore, the position of water molecule W2013 (equivalent to W364 in 2G9C) was taken into account to model the ligand. Compared to W364, W2013 is shifted away from the ligand by 0.75 Å. This indicates that in the complex with **26** a nonhydrogen bonding group occupies the space between this water molecule and the 4-oxo group of U74. Accordingly, the methyl group of **26** was placed into this position. In the resulting binding mode, hydrogen bonds are formed between the amino groups and two of the ring nitrogen atoms of the ligand and the bases of U74 and U51 (all ≤2.9 Å). The distance from the methyl group of the ligand to W2013 is 3.3 Å and to the 4-oxo group of U74 is 3.1 Å. Furthermore, a water molecule (W2216) is found in the pocket which mediates a contact between the ligand and the 2′ hydroxyl group of U22. This water molecule was not observed previously in any other GRA crystal structure. The binding mode of **26** was not predicted by the docking calculations that placed the methyl group of the ligand close to the 4-oxo group of U51 (rmsd = 2.8 Å). If the shifted position of W2013 is taken into account during docking, the correct binding mode is predicted (rmsd = 0.46 Å).

Ligand **27** adopts two distinct binding modes in the GRA binding site (Figure 4C). The ratio of these binding modes is 70:30 as estimated by crystallographic *B*-factors. In both orientations the 2-amino group forms hydrogen bonds with the 2-oxo groups of U74 and U51. In the higher populated binding mode the 2-amino group of the ligand interacts with the 4-oxo group of U51 and water molecule W2149 that was found in a similar position as W2216 in the complex with **26** (Figure 4B). In the alternative binding mode, the 4-amino group hydrogen bonds to the 4-oxo group of U74. No significant electron density was observed for water molecule W364. This is probably due to the multiple binding modes. The ligand could only interact with this water molecule in one of them. In the highest scoring binding mode the ligand orients its 4-amino group toward U51 (rmsd = 0.53 Å). The alternative binding mode differs in score by only 1.5 kJ/mol (rmsd = 0.74 Å).

## Discussion

Modeling of RNA-ligand interactions is relatively unexplored compared to protein-ligand interactions (Fulle and Gohlke, 2010). In particular, there is a dearth of studies that make prospective predictions that are subsequently tested in biochemical assays and by structure determinations. It is often these situations that expose the strengths and weaknesses of the applied methods (Kolb and Irwin, 2009). In the current study, we used a small model binding site to probe RNA-ligand docking, both retro- and prospectively. The simplicity of the GRA binding site allowed us to separate factors that are specific to RNA-ligand docking from more generic molecular docking factors. Four points stand out from this study: (1) A performance similar to protein-ligand docking was achieved using a standard protein-ligand docking program with only minor changes. (2) Molecular docking was able to predict new ligands, some of them based upon scaffolds not previously known to bind to adenine-sensing riboswitches. (3) None of the problems encountered in this study was specific to RNA-ligand docking. Instead, well-known problems in protein-ligand docking such as the role of water molecules, multiple binding modes and variation in protonation states hampered the predictions. (4) GRA was established as a model system for RNA-ligand docking allowing both retro- and prospective predictions. An expansion of these points follows.

We chose a docking program with a physics-based scoring function to probe RNA-ligand docking. In theory, the use of force-field parameters optimized for RNA atoms instead of protein atoms should be sufficient to adapt this scoring function for RNA-ligand docking. However, in terms of molecular recognition there are two main differences between proteins and RNA for which the scoring function might not be appropriate: (1) RNA molecules are highly charged and (2) RNA-ligand interactions are dominated by polar contacts (Hermann, 2000). We addressed the first issue by implicitly modeling charge screening by counter ions. The partial charges of the phosphate oxygen atoms were modified to result in a net charge of 0 per phosphate group as done previously (Detering and Varani, 2004; Moitessier et al., 2006). To address the second issue, we expected that it would be necessary to introduce correction factors to model the balance between E_vdw_, E_elec_, and ΔG_solv_ (Equation 1) correctly. To our surprise, it turned out that this was not the case. By using appropriate parameters for RNA atoms and implicitly modeling counter ions DOCK 3.5.54 was already able correctly to predict all known binding modes of the ligands in the test set and to separate ligands from decoys (Table 1). Admittedly, only moderate correlation between binding affinities and docking scores was obtained. While this is unsatisfactory it is comparable with the performance of protein-ligand scoring functions commonly used for virtual screening (Li et al., 2010a). When docking a large database into the GRA binding site almost perfect discrimination between known ligands and database compounds was obtained (AUC of the ROC curve = 0.98, Figure 3). This is comparable or better to that obtained in protein-ligand docking studies with binding sites of similar complexity as the GRA binding site (Wei et al., 2002; Brenk et al., 2006). Collectively, the retrospective docking results demonstrated that RNA-ligand docking with the chosen parameterization performs well for the GRA binding site. The terms in the physics-based scoring function were well balanced and no additional corrections were necessary in order to apply DOCK 3.5.54 to RNA-ligand docking.

Five high scoring compounds were selected for prospective tests. Four of them were shown experimentally to bind to GRA (Table 3). The most potent of these ligands was **27**. The binding affinity of this compound (*K_D_* = 80 μM) was only 4-fold lower than that of the related ligand **2** (Table 1). Weaker binding probably arose from the lack of an amino group resulting in fewer hydrogen bonds between ligand and GRA (Figure 4C). In **26**, one of the amino groups of **2** is replaced by methyl. Thus, **26** not only forms fewer hydrogen bonds in the binding site than **2** but in addition the hydrophobic methyl group does not satisfy hydrogen-bonding interactions with surrounding polar groups (Figure 4B). Despite these unfavorable contacts, the binding affinity of **26** was only slightly lower (*K_D =_* 110 μM) than that of **27**. When the methyl group of **26** was replaced by a chlorine atom, binding was diminished (**25**, Table 3) There are two probable reasons: (1) If **25** were bound to the receptor in a similar binding mode as **26** or **27**, the potential locations for the chlorine atom would result in close contacts to oxo groups from the surrounding bases. However, the resulting directionality would not satisfy the angular requirements for a C-Cl⋅⋅⋅O interaction, generating repulsion (Bissantz et al., 2010). (2) Due to inductive effects the ring nitrogen atoms of **25** are less basic than those of **26**, leading to weaker hydrogen bonds in the orientation required for riboswitch binding. Obviously, the scoring function was not able to reproduce these effects. Compounds **24** and **28** are both based upon scaffolds that were not previously observed to bind to adenine-sensing riboswitches. With binding affinities of 370 and 650 μM, they are rather weak ligands. The thiadiazole derivative **28** is the smallest GRA or AR ligand known to date. Due to its small size it has only limited shape complementarity with the binding site, which might lead to weak binding properties. Superimposing the binding modes of the ligands **2** and **24** reveals that both compounds have a similar arrangement of hydrogen-bond donor and acceptor groups with respect to U51 and U47 (Figure 4D). However, **24** forms an additional hydrogen bond to the ribose of U47 but is in turn lacking a hydrogen-bond donor group to interact with the 4-oxo group of the base of U74. The difference in the binding affinities of 2,6-diaminopurine (**1**) and 2-aminopurine (**3**) suggests that the formation of the hydrogen bond to U74 can lead to a 30-fold improvement in binding affinity (Table 1) (Gilbert et al., 2006b). Apparently, the additional hydrogen bond formed by **24** with the ribose can not compensate for the loss of this important hydrogen bond. The small size of the GRA binding site combined with the high density of hydrogen-bond donor and acceptor groups leaves little opportunity to derive tightly-binding scaffolds other than purine. Nevertheless, retrieving the ligands **24** and **28** underlines the power of molecular docking to discover hits that are structurally unrelated to known ligands. In previous studies with purine riboswitches, ligands that closely resembled the natural ligands were designed (Kim et al., 2009; Mulhbacher et al., 2010). Clearly, RNA-ligand docking has the potential to exploit the wealth of riboswitch crystal structures to derive ligands that go beyond such close modifications.

The problems that were encountered in this docking study were not specific to RNA docking. Deficiencies of treating structural water molecules hampered the prediction of the binding modes of **14** and **26** and the ranking of **14** (Figure 1 and Table 1). This is a well-known problem in molecular docking (Schneider, 2010) and progress has been made to solve it in recent years (Mancera, 2007). However, commonly used scoring functions are still not able to treat displaceable water molecules reliably (Englebienne and Moitessier, 2009). Decoy **6** obtained a score that was too favorable because it was stored as a neutral molecule in the database whereas the compound is likely to be predominantly deprotonated under assay conditions. We used a rule-based approach to generate protonation states and tautomers for the docking database (Mpamhanga et al., 2009). While this is very fast, it has the drawback that the approach is not generic. Compounds containing patterns for which no rules have been defined will necessarily fail as it was the case with **6** in this study. More sophisticated computational methods for pK_a_ prediction are available (Liao and Nicklaus, 2009; Manchester et al., 2010) and can be used for database preparation (Kalliokoski et al., 2009). The challenge will be to correctly model pK_a_ shifts upon ligand binding (Klebe, 2006). Finally, ligand **27** adopts two distinct binding modes when bound to GRA (Figure 4C). Routinely, molecular docking predicts only one binding mode for each ligand. However, multiple binding modes are frequently observed in crystal structures. Computational methods that take these into account have recently been developed but not yet tested on a larger scale (Gorelik and Goldblum, 2008; Stjernschantz and Oostenbrink, 2010). Improved algorithms and scoring functions addressing any issues discussed above will not only improve RNA-ligand docking, but molecular docking in general.

We established the GRA as a model system for RNA-ligand docking studies. A set of known ligands and decoys together with high-resolution crystal structures is a valuable data source for retrospective testing of docking algorithms and scoring functions. Due to the small size and low complexity of the binding pocket it is possible to identify why incorrect predictions have been made, that in turn can suggest directions for future improvements. The competition binding assay (Figure 3) allows determination of binding affinities of predicted ligands in a less time- and RNA-consuming way than in-line probing and ITC assays used previously (Mandal and Breaker, 2004; Gilbert et al., 2006b). Crystallizing GRA is straightforward and complexes with soluble ligands can be obtained routinely to verify predicted binding modes. In the current study, we considered the GRA binding site to be rigid. Recently determined crystal structure complexes with the related guanine riboswitch revealed that the base in position 74 adopts different conformations depending on the bound ligand, suggesting a degree of induced fit (Gilbert et al., 2009). In ongoing work, we are using this information to study how RNA flexibility can be modeled in RNA-ligand docking.

In summary, GRA is a valuable model system for studying RNA-ligand docking, both retro- and prospectively. Using a standard protein-ligand docking program with only minor modifications, new ligands, including some which are structural distinct from known ligands, were identified. Due to the limited size of the GRA binding pocket with a high density of hydrogen-bond donor and acceptor groups modifications of the ligand scaffold lead to compromised hydrogen-bonding capabilities which are associated with a drop in binding affinity. Nevertheless, the current study demonstrated that molecular docking is a promising tool for the discovery of structural diverse RNA ligands.

## Significance

**Protein-ligand docking is an important tool in structure-based drug design. However, despite the structural knowledge of RNA targets, RNA-ligand docking is still in its infancy limiting the drug discovery process for this class of targets. Using a purine riboswitch as a model system, we demonstrate that RNA-ligand docking is performing comparably to protein-ligand docking. Furthermore, we show that using this method ligands based on novel molecular scaffolds can be identified making RNA-ligand docking a suitable tool to explore the wealth of RNA crystal structures for the discovery of new ligands.**

## Experimental Procedures

### Receptor Preparation

GRA (PDB code 2G9C) was used for docking. Hydrogen atoms were added using Sybyl (Tripos). The positions of the hydrogen atoms were minimized using the MAB force field as implemented in Moloc (Gerber Molecular Design) (Gerber and Muller, 1995) while keeping all other atoms rigid. Afterward, all atoms not part of the RNA except of water molecule W364 were removed. To define the ligand binding site a cubic grid was placed 2 Å around the ligand with a grid width of 1.5 Å. Spheres were placed on all grid points not overlapping with the receptor. Partial charges for all RNA atoms except of phosphate oxygen atoms were obtained from the AMBER99 force-field parameter (Wang et al., 2000). The phosphate oxygen atoms were assigned a partial charge higher than the charge stored in the AMBER database to obtain a net charge of zero per nucleotide, thus mimicking charge screening by nonspecific binding of counter ions (Detering and Varani, 2004; Moitessier et al., 2006). Grids to store information about excluded volumes, electrostatic and van der Waals potential, and solvent occlusion were calculated as described earlier (Brenk et al., 2006).

### Preparation of Small Molecules for Docking

Protonation states, tautomers, partial charges, desolvation energies, and low-energy conformations were calculated as described earlier (Mpamhanga et al., 2009).

For the screening, database compounds were selected from our in-house database containing commercially available compounds (Brenk et al., 2008). All molecules fulfilling the selection criteria were subsequently docked into the riboswitch binding site and only compounds that gave a negative van der Waals energy score were retained for the final docking database and analysis.

Where needed, pK_a_ values were calculated using Marvin (Chemaxon) with default settings.

### Molecular Docking

DOCK 3.5.54 was used to dock small molecules into the GRA binding pocket (Lorber and Shoichet, 1998; Wei et al., 2002). Ligand orientations were sampled using the following settings: ligand and receptor bins were set to 0.5 Å, overlap bins were set to 0.4 Å, and the distance tolerance for matching ligand atoms to receptor matching sites ranged from 1.1 to 1.2 Å. Each docking pose that did not place any atoms in areas occupied by the receptor was scored for electrostatic and van der Waals complementarity (Wei et al., 2002) and penalized according to its estimated partial desolvation energy (B. Shoichet, unpublished). For each compound, only the best-scoring database representation (tautomer, protonation state, multiple ring alignment) was stored in the final docking hit list. Rmsds between docked and crystallographically determined binding modes were calculated after superimposing the N3 atoms of U22, 47, 51, and 74 of the crystal structures onto the receptor used for docking.

### RNA Preparation and Purification

RNA was prepared and purified as described (Gilbert et al., 2006a).

### Ligand-Binding Assay

All compounds for binding studies were purchased from Sigma Aldrich with the following exceptions: **24** was obtained from IBS and **28** was from Chemdiv.

Fluorescence spectra were recorded using an SLM-Aminco 8100 fluorimeter. The sample buffer contained 50 mM Tris-HCl (pH 8.3), 100 mM KCl, and 10 mM MgCl_2_. 2-Aminopurine (**3**) spectra were recorded from 330 to 450 nm with an excitation wavelength of 300 nm. The data were corrected for lamp fluctuations and instrumental variations. The spectra were integrated to determine the total amount of 2-aminopurine fluorescence.

The binding constant of **3** was determined by titrating RNA into a solution containing 200 nM of that compound. After each addition of RNA 3 min were allowed to reach equilibrium. Free 2-aminopurine fluorescence was fitted to a simple binding model. A binding constant of *K_L_* = 140 nM was obtained. Competition experiments were carried out by titrating the competition ligand into a solution containing **3** and GRA. A 2-aminopurine spectrum in fluorescence buffer (typically 213 nM) was recorded. GRA was added to a final concentration of 243 nM resulting in 200 nM 2-aminopurine concentration and left to equilibrate for 10 min. Subsequently, the competition ligand was incrementally added to a final concentration of at least 1.2 mM, except for **28** that was titrated to 0.68 mM and **25** to 0.12 mM due to lower solubility. After each addition of ligand 3 min were allowed for equilibration. Data were processed using Microsoft Excel. Free 2-aminopurine fluorescence was fitted to a two complex binding model (Equation 2) with dilution effects were taken into account (Yan et al., 2005).

(2) $$R^{3}+aR^{2}+bR-K_{L}K_{D}R_{tot}=0,$$

where

$$\begin{array}{l} a=L_{tot}+X_{tot}+K_{L}+K_{D}-R_{tot}\\ b=K_{D}L_{tot}+K_{L}X_{tot}+K_{L}K_{D}-R_{tot}\left(K_{L}+K_{D}\right) \end{array}$$

In these equations, *R* is the concentration of unbound RNA; *R_tot_* the total RNA concentration; *L_tot_* the total 2-aminopurine concentration; *X_tot_* the total concentration of test compound; *K_L_* the dissociation constant of 2-aminopurine; and *K_D_* the dissociation constant of the test compound. All parameters except *R* and *K_D_* are known. *R* was obtained from the fluorescence reading using the known *K_L_*. Experimental error will result in $R^{3}+aR^{2}+bR-K_{L}K_{X}R_{tot}=\chi$ for each data point. Fitting was performed by minimizing $\Phi =\Sigma \chi^{2}$ through varying *K_D_.* The obtained binding constants were rounded to a precision of 0.01 mM (0.001 mM in the case of **5**). The reported binding constants are the average of three measurements.

### Structure Determination

RNA-ligand complexes were crystallized using micro seeding as described earlier. (Batey et al., 2004) For data collection crystals were cryoprotected using the respective well solution with 30% MPD. Cryoprotectant was applied for approximately one minute before freezing in liquid nitrogen. Diffraction data for **14** was collected at Diamond Light Source (Oxford, UK), beamline I03. All other data were collected at beamline ID14-1 at the European Synchrotron Radiation Facility (ESRF) in Grenoble, France. Data were indexed, integrated and scaled using HKL2000 (Otwinowski and Minor, 1997). The structures were solved using MOLREP (Vagin and Teplyakov, 1997) using 2G9C as starting model and refined with REFMAC5 (Vagin et al., 2004) via the CCP4 suite of programs (CCP4, 1994). Ligand topology files were generated using the PRODRG server (Schuttelkopf and van Aalten, 2004). Model building was carried out using COOT (Emsley and Cowtan, 2004).

## Figures and Tables

**Table 1:** Test Set of Experimentally Confirmed Ligands and Decoys Taken from the Literature

Compound #	Rank	Score [kJ/mol] (Rmsd^a^ [Å])	Structure^b^	K_D_ [μM]
**1**	1	−34.18 (0.24)		0.01^c^
**2**	2	−33.97		20^d^
**3**	3	−32.00		0.3^c^
**4**	4	−31.85(0.34)		0.3^c^
**5**	5	−31.81(0.13)		20^d^
**6**	6	−30.44		NAc,e
**7**	7	−29.90		30^c^
**8**	8	−26.71		20^d^
**9**	9	−25.11		NA^c^
**10**	10	−23.24		NA^c^
**11**	11	−21.36		NA^f^
**12**	12	−20.03		NA^c^
**13**	13	−18.99		NA^c^
**14**	14	−18.33		NA^c^
**15**	15	−17.49		100^c^
**16**	16	−15.97		NA^c^
**17**	17	−14.48		NA^c^
**18**	18	−14.17		NA^c^
**19**	19	−11.97		NA^g^
**20**	20	−9.93		NA^g^
**21**	21	−8.50		NA^g^
**22**	22	−7.01		NA^c^
**23**	23	15. 92		NA^c^

a Between crystallographically determined binding mode and highest scoring binding mode.

b Highest scoring database representation (tautomer, protonation state).

c Mandal and Breaker (2004).

d Gilbert et al. (2006a).

e Not applicable.

f Gilbert et al. (2009).

g Batey, unpublished data.

**Table 2:** Crystallographic Data and Refinement Statistics of GRA-Ligand Complexes

Details of Data Collection				
Ligand Complex	**14**	**24**	**26**	**27**
PDB Code	2xo1	2nxw	2nxz	2xo0
Space Group	C2	C2	C2	C2
Unit Cell Dimensions (Å)	a = 132.762	a = 135.895	a = 132.165	a = 130.923
	b = 35.180	b = 35.380	b = 35.068	b = 34.940
	c = 41.763	c = 42.197	c = 41.778	c = 42.074
	β = 90.51	β = 92.20	β = 92.06	β = 92.11
Resolution Range (Å)	20–1.6 (1.66–1.6)	67.88–1.5 (1.55–1.5)	60.08–1.6 (1.66–1.6)	65.5–1.7 (1.76–1.7)
Observations	72,587	80,100	93,460	62,872
Unique Observations	25,098	30,250	25,334	21,077
Completeness (%)	96.5 (91.0)	93.1 (97.9)	98.5 (98.1)	96.8 (93.5)
<I/σ(I) >	20.6 (2.7)	18.0 (2.3)	30.2 (2.9)	26.8 (3.1)
R_merge_^a^ (%)	5.3 (25.8)	6.2 (38.5)	4.4 (39.8)	5.2 (39.1)

**Refinement Statistics**				

Resolution Range (Å)	20–1.6	67.88–1.5	66.08–1.6	65.51–1.7
R-factor^b^ % (R_work_/R_free_)	19.5/21.1	21.4/24.4	19.8/23.4	21.6/27.1
Number of atoms^c^	1364/11/226/56	1366/10/329/54	1382/9/216/52	1363/16/151/55
Mean *B*-factor^d^ (Å^2^)	22.7/18.3/29.0/25.8	21.7/22.1/29.0/23.8	24.9/22.7/29.2/27.9	29.6/26.3/30.2/32.7
RMS bond length deviation (Å)	0.008	0.009	0.009	0.013
RMS bond angle deviation (°)	1.508	1.683	1.546	1.973

Values in brackets are for the highest resolution shell.

a R_merge_ = ∑|I – < I > | / ∑ < I >.

b R-factor = ∑F_o_ – F_c_| / ∑ F_o_.

c Number of atoms.

d Mean *B*-factors for RNA, ligands, water molecules, and other atoms, respectively.

**Table 3:** High Scoring Compounds Selected for Experimental Evaluation

Compound #	Rank	Structure	K_D_ [μM]	Rmsd^a^ [Å]
**24**	3		370 ± 40	0.21
**25**	7		NA^b^	NA.
**26**	11		110 ± 30	0.29
**27**	14		80 ± 20	0.65/0.23
**28**	23		650 ± 180	NA

a Between crystallographically determined binding mode and highest scoring pose.

b Not applicable.

## Accession Numbers

Coordinates have been deposited in the PDB with accession codes 2xo1, 2nxw, 2nxz, and 2xo0.
